# *In Vitro* Activity of Gentamicin-Loaded Bioabsorbable Beads against Different Microorganisms

**DOI:** 10.3390/ma6083284

**Published:** 2013-08-05

**Authors:** Eric Thein, Ulrika Furustrand Tafin, Bertrand Betrisey, Andrej Trampuz, Olivier Borens

**Affiliations:** 1 Department of Orthopaedics and Traumatology, Lausanne University Hospital, Rue du Bugnon 46, Lausanne CH-1011, Switzerland; E-Mails: hanna.furustrand@chuv.ch (U.F.T.); olivier.borens@chuv.ch (O.B.); 2 Department of Infectious Diseases, Lausanne University Hospital, Lausanne, CH-1011, Switzerland; E-Mail: bertrand.betrisey@chuv.ch; 3 Center for Musculoskeletal Surgery, Charité-University Medicine, Free and Humboldt-University of Berlin, Berlin D-10117, Germany; E-Mail: andrej.trampuz@gmail.com

**Keywords:** osteomyelitis, bone graft substitute, antibiotic-loaded bioabsorbable beads, microcalorimetry

## Abstract

Osteomyelitis is responsible for high treatment costs, long hospital stays, and results in substantial morbidity. Treatment with surgical debridement and antibiotic-impregnated Polymethylmetacrylate (PMMA) beads is the standard of care, providing high local but low serum antibiotic concentrations, thereby avoiding systemic toxicity. However, for several reasons, the beads require surgical removal. Alternative antibiotic delivery systems should improve the treatment of bone infection, actively encourage bone healing and require no additional surgery for removal. We investigated the activity of gentamicin-loaded bioabsorbable beads against different microorganisms (*Staphylococcus epidermidis*, *S. aureus*, *Escherichia coli*, *Enterococcus faecalis*, *Candida albicans*) commonly causing surgical site bone infection, by microcalorimetry. Calcium sulphate beads containing gentamicin were incubated in microcalorimetry ampoules containing different concentrations of the corresponding microorganism. Growth medium with each germ and unloaded beads was used as positive control, growth medium with loaded beads alone as negative control. Bacterial growth-related heat production at 37 °C was measured for 24 h. Cultures without gentamicin-loaded beads produced heat-flow peaks corresponding to the exponential growth of the corresponding microorganisms in nutrient-rich medium. In contrast, cultures with gentamicin-loaded beads completely suppressed heat production during 24 h, demonstrating their antibiotic activity. Gentamicin-loaded beads effectively inhibited growth of susceptible microorganisms, under the described *in vitro* conditions.

## 1. Introduction

Perioperative antimicrobial prophylaxis, improved surgical technique and laminar airflows in the operating rooms have diminished the infection rate after internal fixation. However, it still remains approximately 0.5%–2% in closed fractures [1], and up to 30% in Grade III open fractures [2]. This is due to the risk of the implant and the peri-implant tissue becoming infected by pathogens or opportunistic bacteria [3,4]. As the number of implanted devices is constantly rising, the absolute number of implant-related infections is also increasing [5], and is, together with osteomyelitis, responsible for high treatment costs, long hospital stays, and often result in substantial morbidity [6].

The standard treatment of osteomyelitis is a combination of surgical debridement and antimicrobial therapy [7]. Conventional systemic delivery of antibiotics is problematic because of its systemic toxicity with associated renal and liver complications [8,9] and poor antibiotic penetration into ischemic and necrotic tissue (often present in osteomyelitis). In addition, bacteria present in biofilms in the bone or on the implant can be up to 1000 times more resistant to most antimicrobial substances than their planktonic counterparts [10]. Local delivery of antibiotics was already initiated in the 1930s with the appearance of the sulfonamides, but with the arrival of Polymethylmetacrylate (PMMA), Buchholz and Engelbrecht [11] established in the 1970s, the principle of using antibiotic-impregnated bone cement to increase local antibiotic concentration, and thereby enhancing systemic antimicrobial therapy. In 1976, Marks *et al.* [12] proved that oxacillin, cefazolin and gentamicin are each released from acrylic bone cement in a microbiologically active form, and Elson *et al.* [13] showed that if depot PMMA antibiotics are introduced in bone, their concentration in bone is much higher than what can be achieved by safe intravenous administration.

Unfortunately, several concerns about the use of PMMA as a delivery vehicle for depot antibiotics have risen. Retained PMMA acts as a foreign body once the release of the antibiotic has fallen below therapeutic levels, and the presence of prolonged subtherapeutic levels increases the risk of emergence of bacteria resistant to the antibiotic [14]. In addition, the porous PMMA represents an optimal surface for bacterial biofilm formation [15]. Moreover, in situations where large bony defects remain after the debridement, filling these defects with PMMA prevents the bone from healing. Removal of the PMMA depot, with subsequent bone grafting where necessary, is therefore generally advocated as soon as the infection is controlled, requiring a second surgical intervention [16].

Alternative materials to PMMA for the delivery of depot antibiotics should therefore exhibit a faster and more complete release of the contained antibiotic to diminish the risk of resistance development [17]. In addition, the material should be biodegradable or capable of being incorporated into the regenerating bone in order to avoid another surgical intervention for their removal. Bone graft substitutes have already been widely investigated as their implantation combines the possibility to deliver local antibiotics at high concentrations and simultaneously participate in the bone regeneration process [18,19]. Calcium sulphate (CaSO_4_) has been used since 1892 as a bone defect filler [19,20,21], preventing the ingrowth of soft tissue. It provides an osteoconductive matrix for the ingrowth of blood vessels and osteogenic cells that will reabsorb the CaSO_4_ while forming new bone [22,23]. It is probably the most frequent bone graft substitute used in the clinical setting of bone infection treatment, also because it is commercially available under different forms. Several authors already demonstrated the *in vitro* antibacterial activity of gentamicin-loaded plaster of Paris beads on bacterial cultures [24,25]. In a study from Sulo [26] gentamicin-loaded plaster beads were used since 1984 as a bone void filler in 409 patients with chronic osteomyelitis, with 96.5% of them cured, and a filling of bone loss of 50% and more occurring in 81.5%. In these studies the beads were fabricated in an artisanal fashion, the gentamicin powder being hand-mixed into the plaster, a time-consuming and inexact procedure. Several *in vitro* and *in vivo* studies have been performed with commercially available tobramycin-laden calcium sulphate beads [27,28,29], with a high efficacy rate. To our knowledge, no study investigating the antibacterial effect of a commercially available, gentamicin-loaded calcium-sulphate bone graft substitute has been published so far, although gentamicin has been, and still is, a widely used antibiotic for local delivery in the treatment of bone infection [30,31,32].

## 2. Objectives

We investigated the *in vitro* activity of gentamicin-loaded bioabsorbable beads against different microorganisms commonly causing surgical site bone infection, by microcalorimetry. We expect that these beads will exhibit a strong antibacterial local effect against microorganisms susceptible to gentamicin, whereas the growth of germs not susceptible to gentamicin should not be affected.

## 3. Results and Discussion

**Figure 1:**
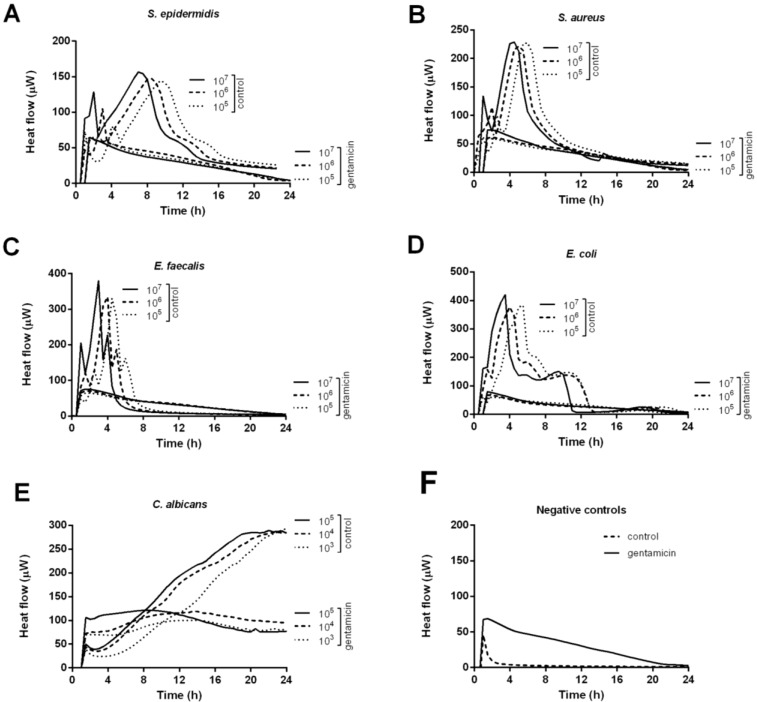
Thermokinetic profiles of *S. aureus* (**A**); *S. epidermidis* (**B**); *E. faecalis* (**C**); *E. coli* (**D**) and *C. albicans* (**E**) in the presence of calcium-sulphate beads with or without (positive control) gentamicin. Beads in growth media without microorganisms served as negative control (**F**).

Figure 1 illustrates the heat produced by *S. aureus* (A), *S. epidermidis* (B), *E. faecalis* (C), *E. coli* (D) and *C. albicans* (E) in the presence of calcium-sulphate beads with or without (control) gentamicin for 24 h.

Beads were challenged with exponentially growing bacteria in concentrations of 10^5^, 10^6^ and 10^7^ CFU/mL. An initial heat-flow was detected for all microorganisms (heat flow exceeding 20 μW) within 1 h independently of the presence of gentamicin. Nevertheless, as shown in Figure 1F, the initial heat is produced from a chemical reaction between the calcium-sulphate bead and the growth media, and is not a result of bacterial growth. A heat-flow peak of 68 μW and 45 μW, was observed for the gentamicin-loaded and the control bead (negative controls), respectively. Whereas the heat-producing reaction between the unloaded control bead and the media only lasted 4 h, the reaction between the gentamicin-loaded bead and the media continued for 24 h.

By including a gentamicin non-susceptible microorganism in the study, *C. albicans*, heat produced by microbial growth could be distinguished from the background heat produced from the chemical reaction between the bead and the media. As shown in Figure 1E, *C. albicans* continued to produce heat over 24 h with a stable heat signal of ~80–100 μW. In contrast, for the different gentamicin-susceptible bacterial species tested, the heat-production decreased during 24 h, indicating that the bacteria were not able to grow (Figure 1A–D). The gentamicin-loaded beads were able to inhibit the highest bacterial load tested (10^7^ CFU/mL) for all bacterial species tested. Control beads exhibited no antibacterial effect. In the presence of an unloaded control bead the bacterial heat-flow continued to rise exponentially until the heat-flow peak was reached. The heat-flow peak is reached when the microorganisms stop to actively divide and enter the stationary growth-phase, due to the lack of nutrients or oxygen.

Table 1 describes the heat-flow peaks for the tested microorganisms in the presence of a control bead or a gentamicin-loaded bead, and the time-to-peak for the controls.

**Table 1:** Heat-flow peaks for tested microorganisms in the presence of gentamicin-loaded or control calcium-sulphate beads.

Heat-flow peaks	Control						Gentamicin		
	Peak (μW)			Time to peak (h)			Peak (μW)		
*Concentration (cfu/mL)*	10^7^	10^6^	10^5^	10^7^	10^6^	10^5^	10^7^	10^6^	10^5^
*S. epidermidis*	111	102	98	7.1	8.2	9.4	0	0	0
*S. aureus*	184	175	183	4.4	4.9	5.7	8	0	0
*E. faecalis*	335	285	285	3.1	4.1	4.6	8	3	0
*E. coli*	375	325	338	3.4	3.9	5.2	12	0	0
*C. albicans* *	249	240	239	24	24	24	53	51	32

* Fungal concentrations tested were 10^5^,10^4^ and 10^3^ CFU/mL.

The heat-flow peak of the negative controls was deduced for the control and the gentamicin beads, respectively. For control beads, a proportional delay in time-to-peak was observed with decreasing bacterial concentrations. When comparing the heat-flow peaks, corresponding to the total number of active cells, a nearly 100% reduction was observed in the presence of gentamicin in comparison to the control beads, for *S. epidermidis*, *S. aureus*, *E. faecalis* and *E. coli*, respectively. Only in the presence of the highest bacterial inoculum some heat was produced. In contrast, for *C. albicans* heat-flow peaks ranging from 51 to 53 μW were observed (after substracting the background heat produced by the bead alone.)

Microcalorimetry is an innovative method based on measuring heat from replicating microorganisms in culture. It was first evaluated as a rapid and accurate screening method for contaminated platelet concentrates [33]. The heat flow is proportional to the quantity of bacteria and can be used for quantitative assessment [34]. For planktonic bacteria, it is now widely used as a non-invasive [35] monitoring instrument due to its high sensitivity, reproducibility and simplicity [36].

In our experiment measures obtained by microcalorimetry indicated a nearly 100% reduction of the heat-flow peaks for all the bacterial species in the presence of gentamicin, in comparison to the control beads, which proved that the gentamicin-loaded beads effectively inhibited even the highest bacterial load tested for all the gentamicin-susceptible bacterial species they were challenged with, clearly demonstrating their antibiotic activity. Our findings correlate thus with the results of Mackey *et al.* [24] and Mousset *et al.* [25], who already demonstrated the *in vitro* antibacterial activity of gentamicin-loaded plaster of Paris beads on bacterial cultures.

Interestingly, when comparing the heat-flow peak of *C. albicans* reached in the presence or absence of gentamicin, a clear reduction in growth-related heat was observed in the presence of the antibiotic-loaded bead, although *C. albicans* is a gentamicin non-susceptible microorganism. As shown by Wichelhaus *et al.* [32] and by Kluin *et al.* [37], calcium-sulphate carriers normally exhibit an *in vitro* burst-release, mostly caused by diffusion, of gentamicin within the first 24 h (approximately 80%), followed by a more gradual release over a period of at least 10 days. Considering the gentamicin loading of 2.5 mg per bead, gentamicin concentrations above 500 µg/mL could be expected in the calorimetric ampoule. Short-duration high-concentration levels at the infection site are desirable for antibiotics such as aminoglycosides that increase their effectiveness with increasing concentration (dose-dependent killing of bacteria) [38]. As gentamicin serum levels above 12 µg/mL are considered toxic [39] (when monitoring therapeutic drug levels), the observed decrease in heat produced by *C. albicans* is most likely induced by the high gentamicin concentration in the growth media. This could become a major issue in the clinical setting, as local antibiotic delivery vehicles diminish systemic toxicity at the price of higher local toxicity [40]. Isefuku *et al.* [41] demonstrated that high local concentrations of antibiotics may alter the bone regeneration process, with gentamicin concentrations exceeding 100 µg/mL significantly decreasing alkaline phosphatase activity and ^3^H-thymidine incorporation, while concentrations above 700 µg/mL also decreased total DNA in a study on human osteoblast-like cells. Hanssen *et al.* [42] postulated that the benefits of high local antibiotic concentrations for infection eradication need to be balanced against the potentially negative effects on the bone regeneration processes which often occupy a considerable place in the treatment of musculoskeletal infection.

Another major issue with local antibiotic delivery vehicles is the fact that there is no correlation between *in vitro* and *in vivo* elution parameters, several studies evaluating calcium sulphate showing considerably longer release duration in animal models than in elution studies [27,43]. For McLaren [17], fluid dynamics in the animal model seem to be more restricted than in the elution bath, and the elution parameters do not accurately reproduce the environment of a wound. Moreover, release characteristics of each preparation of antibiotic delivery vehicle are specific to that preparation. The next step of our investigations will be the determination of the *in vitro* elution kinetics of gentamicin from the investigated calcium sulphate beads in order to verify if the elution profiles are consistent with the ones found by Wichelhaus [32] and Kluin [37], respectively.

Subsequently, standardized *in vivo* investigations that accurately reproduce the clinical wound environment should be carried out, including important variables such as site of implantation (intramedullary or soft tissue), implant size, concentration of contained antibiotic, *etc.*, to determine the expected clinical performance before clinical studies can be initiated.

## 4. Experimental Section

### 4.1. Bone Graft Substitute

Herafill^®^ beads G (Heraeus Medical, Hanau, Germany), biconvex rounded cylindrical beads (250 mg, 6 mm diameter) consisting essentially of calcium sulphate dihydrate, and containing gentamicin (2.5 mg equivalent 1%, in form of gentamicin sulphate), were used. They are indicated to fill bone voids that result from surgical debridement of bone infections, and are supposed to enhance antibiotic protection and encourage bone growth.

Osteoset^®^ beads, obtained from an Osteoset Resorbable Mini-Bead Kit-Fast Cure 5cc (Wright Medical Technology, Arlington, TN, USA), cylindrical beads (100 mg, 4.8 mm diameter) consisting essentially of calcium sulphate hemihydrate and containing no antibiotics, were used in the positive control group (unloaded bead plus microorganism). Osteoset^®^ beads are indicated to be used as osteoconductive material for the ingrowth of blood vessels and osteoprogenitor cells from the graft bed in the treatment of bone voids.

### 4.2. Study Organisms

Laboratory strains of different microorganisms causing bone infection were studied, including *Staphylococcus epidermidis* RP62A (ATCC 35984), *S. aureus* (ATCC 29213), *Escherichia coli* (DH5α), *Enterococcus faecalis* (ATCC 19433) and *Candida albicans* (ATCC 90028). Strains were stored at −80 °C and cultured overnight on human blood agar plates before each experiment.

### 4.3. Evaluation of Gentamicin Activity by Isothermal Microcalorimetry

A bacterial or fungal suspension of McFarland 0.5 was prepared, corresponding to a density of 10^6^–10^8^ colony-forming units (CFU)/mL depending on the microorganism. 4 mL sterile microcalorimetric glass ampoules were filled with 3 mL of Müller-Hinton broth (Sabouraud dextrose broth for *C. albicans*) and inoculated with three different inocula corresponding to final bacterial concentrations of 10^5^, 10^6^ and 10^7^ CFU/mL. *C. albicans* was tested in concentrations of 10^3^, 10^4^ and 10^5^ CFU/mL. Microorganisms were incubated 2 h at 37 °C in order to initiate the exponential growth phase.

After the incubation, one bead, with or without gentamicin, was transferred to each ampoule. An ampoule containing a bead and media only served as negative control. All ampoules were closed with a rubber cap, sealed by manual crimping and sequentially introduced into the calorimetry instrument. After 15 min in the thermal equilibration position, ampoules were lowered into the measurement position. Heat flow was measured for up to 24 h at 10 s intervals. A 48-channel batch calorimeter (thermal activity monitor, model 3102 TAM III; TA Instruments, New Castle, DE, USA) was used to measure the heat flow at 37 °C controlled at 0.0001 °C and a sensitivity of 0.2 μW. Heat was measured continuously and expressed as heat flow over time (in microwatts (μW)). Calorimetric detection time was defined as the time from insertion of the ampoule into the calorimeter until an exponentially rising heat flow signal exceeding 20 μW was detected.

Data collection was performed using the calorimeter manufacturer’s software. Data was processed in Prism software, version 5.0a (GraphPad Software, La Jolla, CA, USA).

## 5. Conclusions

The gentamicin-loaded bioabsorbable calcium sulphate beads investigated in this study effectively inhibited growth of gentamicin-susceptible microorganisms, under the described *in vitro* conditions. Standardized animal studies are mandatory to determine the elution kinetics and the antibacterial effect of gentamicin under *in vivo* conditions. Finally, clinical studies are needed to demonstrate that treatment against bone infection with the use of commercially available gentamicin-loaded bioabsorbable calcium sulphate beads is a good therapeutic option.
